# Diversity, phylogeny and intraspecific variability of *Paradiplozoon* species (Monogenea: Diplozoidae) parasitizing endemic cyprinoids in the Middle East

**DOI:** 10.1017/S0031182023000446

**Published:** 2023-07

**Authors:** Farshad Nejat, Michal Benovics, Eva Řehulková, Jasna Vukić, Radek Šanda, Cüneyt Kaya, Ali Serhan Tarkan, Asghar Abdoli, Sadi Aksu, Andrea Šimková

**Affiliations:** 1 Department of Botany and Zoology, Faculty of Science, Masaryk University, Kotlářská 2, 611 37 Brno, Czech Republic; 2 Department of Ecology, Faculty of Science, Charles University, Viničná 7, Prague 2 128 44, Czech Republic; 3 Department of Zoology, National Museum, Václavské náměstí 68, Prague 1 110 00, Czech Republic; 4 Faculty of Fisheries, Recep Tayyip Erdogan University, 53100 Rize, Turkey; 5 Department of Basic Sciences, Faculty of Fisheries, Muğla Sıtkı Koçman University, 48000 Menteşe, Muğla, Turkey; 6 Department of Biodiversity and Ecosystem Management, Environmental Science Research Institute, Shahid Beheshti University, Shahid Shahriari Sq. Velenjak, 1983969411 Tehran, Iran; 7 Vocational School of Health Services, Eskişehir Osmangazi University, Büyükdere Meşelik Yerleşkesi, 26040 Eskişehir, Turkey

**Keywords:** Cyprinoidei, evolutionary history, host specificity, parasite fauna, phylogeography

## Abstract

Diplozoidae are common monogenean ectoparasites of cyprinoid fish, with the genus *Paradiplozoon* being the most diversified. Despite recent studies on Diplozoidae from Europe, Africa and Asia, the diversity, distribution and phylogeny of this parasite group appears to be still underestimated in the Middle East. The objective of this study was to investigate the diversity, endemism and host specificity of diplozoids parasitizing cyprinoid fish from the Middle East, considering this region as an important historical interchange of fish fauna, and to elucidate the phylogenetic position of Middle Eastern *Paradiplozoon* species within Diplozoidae. Four *Paradiplozoon* species were collected from 48 out of 94 investigated cyprinoid species. Three known species, *Paradiplozoon homoion*, *Paradiplozoon bliccae* and *Paradiplozoon bingolensis*, were recorded on new cyprinoid host species, and a new species, *Paradiplozoon koubkovae* n. sp., was recorded on *Luciobarbus capito* and *Capoeta capoeta* from the Caspian Sea basin in Iran and Turkey. *Paradiplozoon bliccae*, exhibiting a wide host range in the Middle East, expressed both morphological and genetic intraspecific variabilities. The four *Paradiplozoon* species collected in the Middle East were placed in divergent clades, showing the rich evolutionary history of diplozoid parasites in the Middle East. Our study also revealed that two lineages of African diplozoids have a Middle Eastern origin. We stress the importance of applying an integrative approach combining morphological, ecological and molecular methods to reveal the real diversity of diplozoids.

## Graphical abstract

**Figure:**
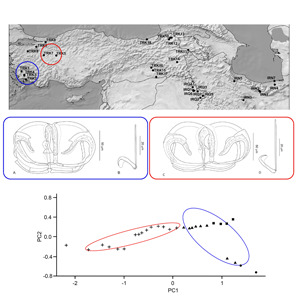

## Introduction

Diplozoidae Palombi, 1949 is a group of monogenean ectoparasites that mostly infect cyprinoid fishes (Pugachev *et al*., 2009). This group is represented by two subfamilies – Diplozoinae Palombi, 1949 and Neodiplozoinae Khotenovsky, 1981. The typical feature of the members of Diplozoidae is the cross-shaped pattern of mature specimens, i.e. the adult body is formed by two permanently fused specimens (Khotenovsky, 1985*a*, 1985*b*; Pugachev *et al*., 2009). While the adult of Diplozoinae species has four pairs of clamps as parts of two attachment organs, the adult of Neodiplozoinae is equipped with more than four pairs of clamps. The Neodiplozoinae include two genera – *Afrodiplozoon* Khotenovsky, 1981 and *Neodiplozoon* Tripathi, 1960, both reported only from Africa (Khotenovsky, 1985*b*). The Diplozoinae include the following five genera: *Diplozoon* von Nordmann, 1832; *Eudiplozoon* Khotenovsky, 1984; *Inustiatus* Khotenovsky, 1978; *Paradiplozoon* Akhmerov, 1974 and *Sindiplozoon* Khotenovsky, 1981, all with a distribution previously considered to be limited to Palaearctic and Oriental zoogeographical ecoregions (Khotenovsky, 1985*b*; Pugachev *et al*., 2009). However, *Paradiplozoon* species have recently been described also from the African continent (e.g. Avenant-Oldewage *et al*., 2014; Dos Santos *et al*., 2015; Benovics *et al*., 2021). Adult diplozoids are located on fish gills and are obligatory blood-feeders, with some species, e.g. *Eudiplozoon nipponicum*, being important pathogens of cyprinoid fishes (Smyth and Halton, 1983; Khotenovsky, 1985*a*; Pugachev *et al*., 2009; Rohlenová *et al*., 2011). Diplozoids are hermaphrodites with a monoxenous life cycle and usually lay one egg at a time, from which a free-living larva (oncomiracidium) hatches and actively seeks a host. After attaching to the host body, larvae move to the gills and grow into their post-oncomiracidial stage, termed diporpa. Two diporpae in their last stage of development (with three or four pairs of clamps already developed) fuse, and after reaching sexual maturity, copulate (Khotenovsky, 1985*a*; Pugachev *et al*., 2009). The vitellarium, pharynx and suckers are located in the anterior parts of the fused specimens, and the haptor (comprising sclerotized hard clamps and central hooks), testes and ovaries are located in the posterior parts of the bodies of the fused specimens (Khotenovsky, 1985*a*). While the clamps are the main attachment features in mature diplozoids, central hooks are used by the oncomiracidium for attaching to hosts (Bychowsky and Nagibina, 1959; Khotenovsky, 1985*a*). The shapes and sizes of individual parts of the attachment apparatus are considered as the taxonomically most important characteristics, used for the species identification of diplozoids; however, some studies showed that the size of the clamps may correlate with the size of the host fish (e.g. Matějusová *et al*., 2002). Therefore, it has been suggested that the shape of individual haptoral sclerites represents the most important taxonomical feature for diplozoid identification (e.g. Matějusová *et al*., 2001*b*, 2002; Dos Santos and Avenant-Oldewage, 2020).

There are also a few records of diplozoids on other fish species such as African Alestidae (Thomas, 1957; Paperna, 1969, 1979; Echi and Ezenwaji, 2010), Cichlidae (Batra, 1984; Yildirim *et al*., 2010), Gobiidae (Khotenovsky, 1985*b*) and Cottidae (Nicoll, 1924; Khotenovsky, 1985*b*). Different levels of host specificity have been reported for diplozoid species. Some species are true generalists (i.e. species infecting a wide range of host species), e.g. *Paradiplozoon homoion* (Khotenovsky, 1985*b*; Gelnar *et al*., 1994; Matějusová *et al*., 2002) and *Paradiplozoon megan* (Benovics *et al*., 2021); however, most diplozoid species have been considered to be strictly host specific, e.g. *Paradiplozoon moroccoensis* (Benovics *et al*., 2021), *Paradiplozoon bingolensis* (Civáňová *et al*., 2013) and *Paradiplozoon iraqensis* (Al-Nasiri and Balbuena, 2016), as they are each currently reported only from a single-host species. In some studies, host specificity is applied as a taxonomically important criterion to describe new diplozoid species (e.g. Al-Nasiri and Balbuena, 2016), which has strongly biased new taxonomical reports. Although most published studies focused on diplozoids have been of a strictly taxonomic nature, i.e. aimed only at describing new species (e.g. Avenant-Oldewage *et al*., 2014; Dos Santos *et al*., 2015; Fan *et al*., 2018; Arken *et al*., 2021; Cao *et al*., 2022) or re-describing previously known ones (e.g. Jirsová *et al*., 2018, 2021; Přikrylová *et al*., 2018), the taxonomy of Diplozoidae is still problematic. Only a few recently published studies have tackled taxonomic issues relating to Diplozoidae by also employing a molecular phylogenetic approach in combination with morphological data; however, they unanimously suggest a need for major taxonomical revision within Diplozoidae (Civáňová *et al*., 2013; Přikrylová *et al*., 2018; Dos Santos and Avenant-Oldewage, 2020; Benovics *et al*., 2021).

For the Middle East region, there are some generic checklists and faunistic reports that lack comprehensiveness and suggest that Iranian and Iraqi parasite faunas are only weakly investigated (e.g. Pazooki and Masoumian, 2012; Abdul-Ameer and Mhaisen, 2013; Al-Nasiri, 2017; Mhaisen and Abdullah, 2017; Mhaisen, 2019); they reference outdated studies, do not investigate all regions and host taxa and often fail to consider the taxonomical reclassification of hosts. On the basis of the freshwater parasite fauna studies of fish in Turkey, 60 monogenean species were reported from native fish species (Öktener, 2003, 2014; Cinar, 2014). However, with respect to the diversity and distribution of Diplozoidae, the Middle Eastern region is only poorly investigated, with only a few studies focusing on Diplozoidae species diversity and the phylogenetic position of diplozoid species parasitizing native cyprinoids in Turkey (Civáňová *et al*., 2013; Unal *et al*., 2017).

To help fill this knowledge gap, the present study was designed (1) to investigate the distribution and diversity of diplozoids on cyprinoid host species in the Middle East, hypothesizing that endemic host species harbour endemic parasites; (2) to reveal the degree of host specificity in Diplozoidae species in the Middle East in light of high cyprinoid endemism in the region and (3) to investigate the position of Middle East species within diplozoid phylogeny, since the Middle East is considered as a historical crossroads for cyprinoid fishes (Coad, 1996; Durand *et al*., 2002).

## Materials and methods

### Sampling and species identification

**Figure 1.**
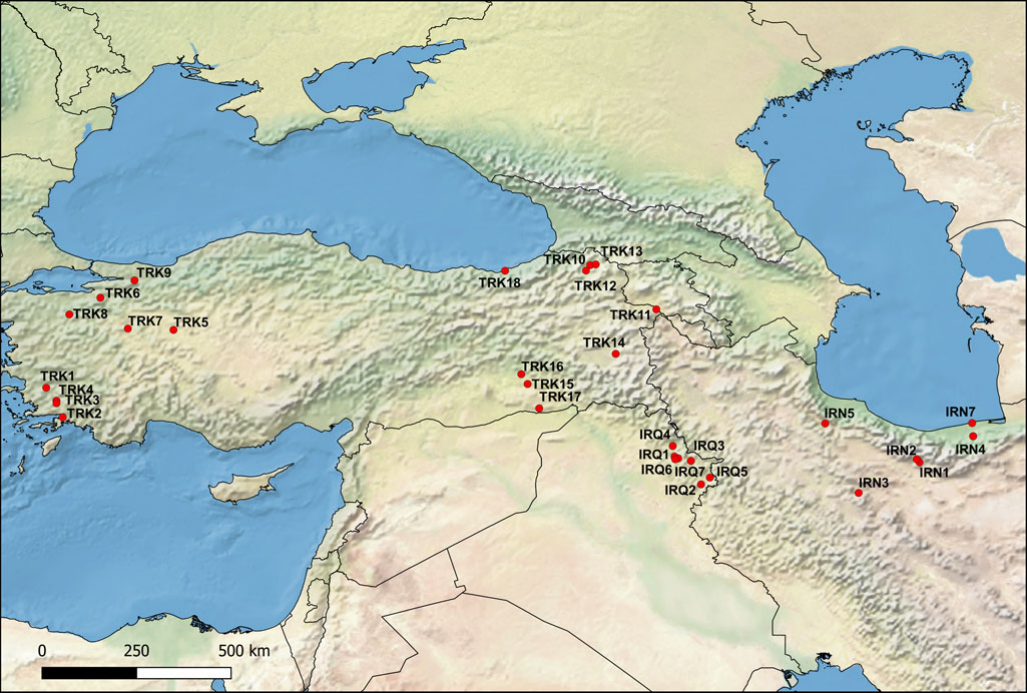
Distribution map of diplozoid species collected during this study. Codes used in the map correspond to the locality codes in Table 1. IRQ: Iraq, IRN: Iran, TUR: Turkey

From 2018 to 2022, a total of 794 fish specimens were sampled at 32 localities in Iraq, Iran and Turkey using electrofishing (Supplementary Table 1, and Fig. 1). A total of 93 endemic cyprinoid species and one non-endemic species (*Vimba vimba*) were examined for the presence of diplozoid monogeneans (list of parasitized species is shown in Table 1). The species identification of the fish hosts was performed by means of morphology using relevant literature keys, and molecular data (i.e. complete cytochrome *b* sequences following Šanda *et al*. (2008)). The parasitological dissection protocol of Řehulková *et al*. (2018) was followed. The parasites were mounted on slides and preserved in a mixture of glycerine and ammonium picrate (GAP; Malmberg, 1957). Prior to mounting, one of the parasite anterior parts was excised using fine needles and preserved in 96% ethanol for further DNA extraction. At least two whole specimens of selected *Paradiplozoon* species were flattened and preserved in 4% formaldehyde, and later stained by iron acetocarmine, dehydrated using a series of graded ethanol concentrations and mounted on slides in Canada balsam (following Georgiev, 1986) to study the internal structures (i.e. soft body parts). Diplozoids fixed in GAP or Canada balsam were identified following available keys and taxonomic papers (Pugachev *et al*., 2009; Civáňová *et al*., 2013; Avenant-Oldewage *et al*., 2014; Dos Santos *et al*., 2015; Al-Nasiri and Balbuena, 2016; Jirsová *et al*., 2018, 2021; Přikrylová *et al*., 2018; Benovics *et al*., 2021) and using an Olympus BX51 light microscope (Olympus, Shinjuku, Japan) equipped with phase contrast.

**Table 1.** List of collected *Paradiplozoon* species including host species, and epidemiological data

Parasite	Host species	*N*	*P*	*I*	*A*	LocID
*Paradiplozoon bingolensis* Civáňová, Koyun & Koubková, 2013	Cyprinion kais * Heckel, 1843	8	37	1–2	0.5 (0.0–1.0)	TRK15
	Cyprinion macrostomum * Heckel, 1843	5	20	3	0.6 (0.0–1.2)	IRQ2
	*C. macrostomum*	9	54	1–2	1.0 (0.3–1.6)	IRQ4
*Paradiplozoon bliccae* (Reichenbach-Klinke, 1961)	Barbus xanthos * Güçlü, Kalayci, Küçük & Turan, 2020	7	43	1–19	5.0 (0.1–11.9)	TRK1
	Capoeta aydinensis * Turan, Küçük, Kaya, Güçlü & Bektaş, 2017	10	40	1–2	0.6 (0.1–1.1)	TRK1
	Luciobarbus kottelatti * Turan, Ekmekci, Ilhan & Engin, 2008	10	100	1–4	2.1 (1.4–2.7)	TRK1
	Petroleuciscus ninae * Turan, Kalayci, Kaya, Bektas & Kucuk, 2018	14	43	1–20	2.9 (0.6–7.6)	TRK1
	*P. ninae*	15	7	1	0.1 (0.0–0.2)	TRK4
	*Squalius fellowesii* (Günther, 1868)	12	8	2	0.2 (0.0–0.5)	TRK1
	*S. fellowesii*	10	90	2–9	3.4 (2.2–5.2)	TRK3
	Ladigesocypris ghigii * (Gianferrari, 1927)	10	50	1–4	1.2 (0.4–2.1)	TRK3
	Vimba mirabilis * (Ladiges, 1960)	10	10	1	0.1 (0.0–0.3)	TRK2
	Vimba vimba * (Linnaeus, 1758)	2	100	1–2	1.5 (1.0–2.0)	TRK5
*Paradiplozoon koubkovae* n. sp.	Capoeta capoeta * (Güldenstädt, 1773)	4	75	1–2	1.3 (1.0–1.7)	TRK11
	Luciobarbus capito * (Güldenstädt, 1773)	3	33	1	0.3 (0.0–0.7)	IRN4
*Paradiplozoon homoion* (Bychowsky and Nagibina, 1959)	Acanthobrama marmid * Heckel, 1843	10	30	1–5	0.7 (0.1–2.1)	IRQ3
	Acanthobrama microlepis * (De Filippi, 1863)	5	40	7–17	4.8 (0.0–11.6)	TRK13
	*A. microlepis*	1	100	1	–	TRK11
	*Alburnoides* aff. damghani * Jouladeh-Roudbar, Eagderi, Esmaeili, Coad & Bogutskaya, 2016	2	100	1	1	IRN5
	Alburnoides eichwaldii * (De Filippi, 1863)	6	33	1–6	1.2 (0.0–0.3)	TRK12
	*A. eichwaldii*	4	25	3	0.7 (0.0–1.5)	TRK10
	Alburnoides emineae * Turan, Kaya, Ekmekçi & Doğan, 2014	10	20	1–2	0.3 (0.0–0.8)	TRK17
	Alburnoides kosswigi * Turan, Kaya, Bayçelebi, Bektaş & Ekmekçi, 2017	10	100	2–5	2.7 (2.0–3.5)	TRK8
	Alburnoides namaki * Bogutskaya & Coad, 2009	4	33	1	0.2 (0.0–0.5)	IRN2
	Alburnus carianorum * Freyhof, Kaya, Bayçelebi, Geiger & Turan, 2019	3	100	2–4	3.3 (2.0–4.0)	TRK2
	*Alburnus chalcoides* (Güldenstädt, 1772)	5	80	1–19	6.6 (0.6–14.0)	IRN2
	Alburnus escherichii * Steindachner, 1897	10	30	1–2	0.4 (0.0–0.9)	TRK7
	*A. escherichii*	10	40	1–3	0.6 (0.1–1.1)	TRK8
	Alburnus filippii * Kessler, 1877	6	50	1–3	1.0 (0.2–2.0)	TRK10
	Alburnus hohenackeri * Kessler, 1877	15	13	1	0.1 (0.0–0.3)	TRK11
	Alburnus sellal * Heckel, 1843	7	43	1–2	0.7 (0.0–1.3)	IRQ7
	*A. sellal*	1	100	2	0.7 (0.0–1.3)	IRQ4
	*A. sellal*	6	67	1–5	1.5 (0.5–3.0)	IRQ5
	*Alburnus* sp.	19	53	1–2	0.6 (0.3–1.0)	IRQ5
	Alburnus timarensis * Kuru, 1980	5	80	1–12	4.4 (1.4–9.0)	TRK14
	Barbus cyri * De Filippi, 1865	4	25	1	0.2 (0.0–0.5)	IRN6
	*Barbus lacerta* Heckel, 1843	10	70	1–13	2.5 (1.0–6.2)	IRQ6
	Barbus miliaris * De Filippi, 1863	5	40	2	0.8 (0.0–1.2)	IRN3
	Barbus oligolepis * Battalgil, 1941	12	8	1	0.1 (0.0–0.2)	TRK6
	Barbus tauricus * Kessler, 1877	10	100	1–13	6.1 (4.0–8.4)	TRK18
	Capoeta alborzensis * Jouladeh-Roudbar, Eagderi, Ghanavi & Doadrio, 2016	16	31	1–5	0.6 (0.2–1.6)	IRN1
	Capoeta buhsei * Kessler, 1877	10	40	1	0.4 (0.1–0.6)	IRN1
	*C. buhsei*	4	100	1–3	2.0 (1.0–2.5)	IRN2
	Capoeta damascina * (Valenciennes, 1842)	2	100	1–2	1.5 (1.0–1.5)	TRK14
	Paracapoeta trutta * (Heckel, 1843)	10	10	1	0.1 (0.0–0.3)	IRQ6
	Chondrostoma regium * (Heckel, 1843)	6	50	1–9	2.8 (0.3–6.2)	IRQ3
	Garra rufa * (Heckel, 1843)	10	20	1	0.2 (0.0–0.4)	IRQ1
	Garra variabilis * (Heckel, 1843)	15	13	1–2	0.2 (0.0–0.5)	TRK16
	Luciobarbus barbulus * (Heckel, 1847)	7	42	1–2	0.6 (0.1–1.0)	IRQ3
	*Luciobarbus mursa* (Güldenstädt, 1773)	5	20	1	0.2 (0.0–0.4)	IRN6
	Petroleuciscus borysthenicus * (Kessler, 1859)	5	20	1	0.2 (0.0–0.4)	TRK6
	Squalius agdamicus * Kamensky, 1901	6	17	1	0.2 (0.0–0.4)	TRK10
	Squalius berak * Heckel, 1843	10	30	1–4	0.7 (0.1–1.8)	IRQ6
	Squalius cii * (Richardson, 1857)	8	100	1–8	4.4 (2.5–6.1)	TRK6
	*S. cii*	5	80	1–3	1.4 (0.4–2.2)	TRK9
	Squalius lepidus * Heckel, 1843	10	50	1–10	1.7 (0.5–4.7)	IRQ3
	Squalius pursakensis * (Hankó, 1925)	7	57	1	0.6 (0.0–0.7)	TRK8
	Squalius turcicus * De Filippi, 1865	7	29	2–3	0.7 (0.0–1.7)	IRN7

P, prevalence in %; I, minimum and maximum intensity of infection; A, mean abundance with confidence interval; N, host sample size; LocID, locality code; asterisk (*), new host records.

### Parasite infection

All epidemiological parameters were calculated following Bush *et al*. (1997). Those were: (1) prevalence, as the proportion of hosts infected by a given parasite species in the whole sample of examined hosts; (2) the intensity of infection, as the number of individual parasites on an infected host and (3) abundance, as the number of individual parasites on a given host regardless of the infection. Following the recommendation of Rózsa *et al*. (2000), confidence levels of 95% of mean abundance were calculated to properly infer epidemiological data. Using QPweb (http://www2.univet.hu/qpweb/qp10/index.php), bias-corrected and accelerated bootstrap confidence intervals were calculated due to the low number of hosts (Reiczigel *et al*., 2019).

### Morphometric data

**Figure 2.**
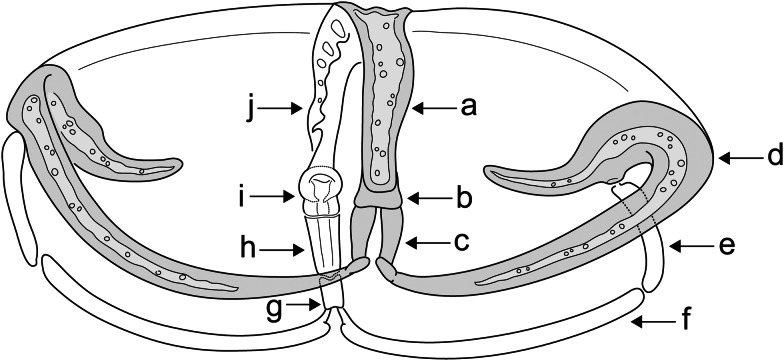
Scheme of general structures of diplozoid clamps: (a) anterior half of median plate; (b) trapeze spur; (c) anterior joining sclerite; (d) anterior clamp jaw; (e) lateral sclerite; (f) posterior clamp jaw; (g) distal posterior joining sclerite; (h) proximal posterior joining sclerite; (i) tendon guiding termination and (j) posterior half of median plate.

Drawings of the attachment clamps and central hooks were made using an Olympus BX51 light microscope equipped with phase-contrast optics and a drawing tube. Drawings were redrawn and digitized using a graphic tablet (Wacom, Kazo, Japan) and Adobe Illustrator CS6 (Adobe, San Jose, USA). Measurements (in *μ*m) were taken using digital image analysis (Stream Motion, version 1.9.2) (Olympus, Shinjuku, Japan) and are given as the mean followed by the range in parentheses (in *μ*m). The terminology for haptoral structures follows that of Owen (1963), Khotenovsky (1985*a*) and Benovics *et al*. (2021) with a little modification (see Fig. 2 for terminology). Type and voucher specimens were deposited in the Helminthological Collection of the Institute of Parasitology (IPCAS), Biology Centre of the Czech Academy of Sciences, České Budějovice, Czech Republic. To comply with the regulations set out in Article 8.5 of the amended 2012 version of the International Code of Zoological Nomenclature (ICZN 2012), details of the new species have been submitted to ZooBank.

In addition, to assess intraspecific morphological variability in the morphometrical characteristics of the haptoral elements of collected *Paradiplozoon* species, the following analyses were performed. To remove the effect of host body size on parasite body size, linear regression was used with the host's standard length as the independent variable and haptoral element measurements as a dependent variable; the residuals from linear regression were used for further analysis. Principal component analysis (PCA) was used to differentiate phenotype variability among selected generalist *Paradiplozoon* species. All statistical analyses were performed and visualized by R v. 4.1.3 using the packages ‘FactoMineR’, ‘ggplot2’, ‘ggbiplot’ and ‘ggpubr’.

### DNA extraction, amplification and sequencing

Prior to DNA extraction, the parasite tissues preserved in 96% ethanol were dried using a vacuum centrifuge. DNeasy Blood & Tissue Kit (Qiagen, Hilden, Germany) was used for extraction following the manufacturer's protocol. Primers D (5′-GGCTYRYGGNGTCGATGAAGAACGCAG-3′) and B1 (5′-GCCGGATCCGAATCCTGGTTAGTTTCTTTTCCT-′3) (Baehellerie and Qu, 1993) were used to amplify the complete internal transcribed spacer 2 (ITS2) region, and the polymerase chain reaction (PCR) amplification protocol following Matějusová *et al*. (2001*b*). For fish tissue, the amplification of the entire cytochrome *b* gene was performed using forward primer GluF (5′-AACCACCGTTGTATTCAACTACAA-3′) and reverse primer ThrR (5′-ACCTCCGATCTTCGGATTACAAGACCG-3′) (Machordom and Doadrio, 2001), following the amplification protocol according to Šanda *et al*. (2008). PCR products were checked on 1% agarose gel and then purified using the ExoSAP-IT enzyme standard protocol (Amplia, Bratislava, Slovakia). Sequencing was carried out at the Macrogen Service Centre (Amsterdam, Netherlands) using the amplification primers. Newly acquired sequences of *Paradiplozoon* specimens and fish hosts were deposited in the GenBank database (see Table 2 for accession numbers).

**Table 2.** List of Diplozoidae species included in phylogenetic analyses

Parasite	Host species	Locality	Country	Parasite's accession number	Host's accession number
*Afrodiplozoon polycotyleus* (Khotenovski, 1981)	*Labeobarbus marequensis* (Smith, 1841)	Lutanandwa River	South Africa	LT719088	NA
*Diplozoon paradoxum* Nordmann, 1832	*Abramis brama* (Linnaeus, 1758)	NA	China	KP326299	NA
	*A. brama*	NA	France	AF369759	NA
	*A. brama*	Kyjovka River	Czech Republic	AJ563372	NA
*Eudiplozoon kamegaii* Nishihira and Urabe, 2020	*Cyprinus carpio* Linnaeus, 1758	NA	Japan	LC517168	NA
	*C. carpio*	NA	Japan	LC517166	NA
	*C. carpio*	NA	Japan	LC517167	NA
*Eudiplozoon nipponicum* (Khotenovsky, 1984)	*Carassius auratus* (Linnaeus, 1758)	Shiga, Takashima, Shin-asahi, Ohta	Japan	LC496174	NA
	*Carassius auratus* (Linnaeus, 1758)	Shiga, Takashima, Shin-asahi, Ohta	Japan	LC496175	NA
	*Carassius auratus* (Linnaeus, 1758)	Shiga, Takashima, Shin-asahi, Ohta	Japan	LC496176	NA
	*C. carpio*	NA	France	AF369758	NA
	*C. carpio*	Morava River	Czech Republic	AJ300710	NA
	*C. carpio*	NA	China	MW025122	NA
*Inustiatus aristichthysi* (Ling, 1973)	*Hypophthalmichthys nobilis* (Richardson, 1845)	Tangxun Lake	China	DQ098894	NA
*Inustiatus inustiatus* (Khotenovsky, 1978)	*Hypophthalmichthys molitrix* (Valenciennes, 1844)	Tangxun Lake	China	DQ098893	NA
*Octomacrum europaeum* Roman & Bychowsky, 1956	*Alburnoides bipunctatus* (Bloch, 1782)	Pasłęka River	Poland	MT441500	NA
*Paradiplozoon barbi* (Reichenbach-Klinke, 1951)	NA	NA	NA	MN688771	NA
*P. bingolensis*	*C. kais*	Sinanköy, Akçayır Stream	Turkey	OP588752*	OQ798014*
	*C. macrostomum*	Darbandikhan Lake	Iraq	OP588753*	OQ797994*
	*C. macrostomum*	Dukan Lake	Iraq	OP588754*	OQ797995*
	*G. rufa*	Goynuk Stream, tributary of the Murat River	Turkey	HE653910	NA
*P. bliccae*	*B. xanthos*	Kamişdere stream, near Yatagan	Turkey	OP588755*	OQ798010*
	*Blicca bjoerkna* (Linnaeus, 1758)	Morava River	Czech Republic	AJ300712	NA
	*C. aydinensis*	Kamişdere stream, near Yatagan	Turkey	OP588756*	OQ798011*
	*Luciobarbus kottelati*	Kamişdere stream, near Yatagan	Turkey	OP588757*	OQ798017*
	*P. ninae*	Kamişdere stream, near Yatagan	Turkey	OP588758*	OQ798018*
	*S. fellowesii*	Doganbaba Creek, Burdur	Turkey	LT560257	NA
	*S. fellowesii*	Kamişdere stream, near Yatagan	Turkey	OP588759*	OP728023
	*L. ghigii*	Kocaalam Deresi	Turkey	OP588760*	OQ798016*
	*V. mirabilis*	Çine River, near Çiftlikköy	Turkey	OP588761*	OQ798022*
	*V. vimba*	Çifteler	Turkey	OP588762*	OQ798023*
*P. koubkovae* n. sp.	*C. capoeta*	B-20 Canal at Aralık, Aras	Turkey	OP588795*	OQ798012*
	*C. capoeta*	B-20 Canal at Aralık, Aras	Turkey	OP588796*	OQ798013*
	*L. capito*	Ghezel Ozan River, Gilvan	Iran	OP588797*	OQ797985*
*Paradiplozoon helleni* Koubková, Benovics & Šimková, 2021	*Scardinius acarnanicus* Economidis, 1991	Trichonis Lake, Panetolio	Greece	MT417731	NA
	*Tropidophoxinellus hellenicus* (Stephanidis, 1971)	Amvrakia Lake, Rivio	Greece	MT417730	NA
*Paradiplozoon hemiculteri* (Khotenovsky, 1985)	*Hemiculter leucisculus* (Basilewsky, 1855)	Zhenjiang, Shaoguan	Zhenjiang, Shaoguan	KY124654	NA
*P. homoion*	*A. marmid*	Du Choman, Aw-e Shiler River	Iraq	OP588763*	OQ797988*
	*A. microlepis*	Ölçek, Ölçeksuyu	Turkey	OP588764*	OQ798000*
	*A. eichwaldii*	Ardahan	Turkey	OP588765*	OQ798001*
	*A. emineae*	Taşlıburç, Çağ-Çağ stream	Turkey	OP588766*	OQ798002*
	*A. kosswigi*	Porsuk Tibet	Turkey	OP588767*	OQ798003*
	*A. carianorum*	Çine River, near Çiftlikköy	Turkey	OP588768*	OQ798004*
	*A. chalcoides*	Jajrood River, Lavasan	Iran	OP588769*	OQ797981*
	*A. escherichii*	Kütahya	Turkey	OP588770*	OQ798005*
	*A. hohenackeri*	Aralik, Aras	Turkey	OP588771*	OQ798006*
	*A. sellal*	Zahrzi, Tabin River	Iraq	OP588772*	OQ797989*
	*A. sellal*	Dukan Lake	Iraq	OP588773*	OQ797990*
	*A. sellal*	Grdi Go, Zalm Stream	Iraq	OP588774*	OQ797991*
	*Alburnus* sp.	Grdi Go, Zalm Stream	Iraq	OP588775*	OQ797992*
	*A. timarensis*	Karasu Stream	Turkey	OP588776*	OQ798007*
	*Barbus haasi* Mertens, 1925	Uldemo River	Spain	MT417728	NA
	*B. cyri*	Tajan River, Alavi Kola	Iran	OP588777*	OQ797982*
	*B. lacerta*	Kani Shok	Iraq	OP588778*	OQ797993*
	*B. oligolepis*	East of Barakfaith, Kurutma kanali, Nilufer	Turkey	OP588779*	OQ798008*
	*B. tauricus*	inflow of Iyidere	Turkey	OP588780*	OQ798009*
	*C. alborzensis*	Jajrood River, Saeed Abad	Iran	OP588781*	OQ797983*
	*C. buhsei*	Jajrood River, Lavasan	Iran	OP588782*	OQ797984*
	*C. regium*	Du Choman, Aw-e Shiler River	Iraq	OP588783*	OQ797997*
	*G. rufa*	Suleymania-Dukan, Little Zab	Iraq	OP588784*	OQ797996*
	*G. variabilis*	Darköprü, Çelebyian Stream	Turkey	OP588785*	OQ798015*
	*L. barbulus*	Du Choman, Aw-e Shiler River	Iraq	OP588786*	OQ797998*
	*L. mursa*	Tajan River, Alavi Kola	Iran	OP588787*	OQ797986*
	*P. borysthenicus*	East of Barakfaith, Kurutma kanali, Nilufer	Turkey	OP588788*	OP728008
	*Telestes beoticus* (Stephanidis, 1939)	Livadia	Greece	MT417729	NA
	*Rhodeus amarus* (Bloch, 1782)	Susurluk Cayi	Turkey	MT028131	NA
	*S. agdamicus*	Ardahan	Turkey	OP588789*	OQ798019*
	*S. berak*	Kani Shok, tributary of Tabin River	Iraq	OP588789*	OQ797999*
	*S. cii*	East of Barakfaith, Burutma kanali, Nilufer	Turkey	OP588791*	OQ798020*
	*S. lepidus*	Du Choman, Aw-e Shiler River	Iraq	OP588792*	OP728025
	*S. cii*	Sapanca, inflow to the Sapanca Lake	Turkey	OP588793*	OQ798021*
	*S. turcicus*	Tajan River, Zarde	Iran	OP588794*	OQ797987*
*Paradiplozoon ibericus* Koubková, Benovics & Šimková, 2021	*Iberochondrostoma lusitanicum* (Collares-Pereira, 1980)	Colares River	Portugal	MT417727	NA
	*Luciobarbus guiraonis* (Steindachner, 1866)	Turia River	Spain	MT417725	NA
	*Parachondrostoma turiense* (Elvira, 1987)	Turia River	Spain	MT417724	NA
	*Squalius pyrenaicus* (Günther, 1868)	Colares River	Portugal	MT417726	NA
	*Squalius valentinus* Doadrio & Carmona, 2006	Magro River	Spain	MT417723	NA
*Paradiplozoon ichthyoxanthon* Avenant-Oldewage, le Roux, Mashego & van Vuuren, 2013	*Labeobarbus aeneus* (Burchell, 1822)	Vaal River	South Africa	HF566124	NA
*Paradiplozoon krugerense* Dos Santos & Avenant-Oldewage, 2016	*Labeo rosae* Steindachner, 1894	Olifants River	South Africa	LT574865	NA
*Paradiplozoon megan* (Achmerov, 1974)	*Squalius cephalus* (Linnaeus, 1758)	Morava River	Czech Republic	AJ300711	NA
	*Squalius squalus* (Bonaparte, 1837)	Cerfone River, Intoppo	Italy	MT417733	NA
	*Squalius zrmanjae* Karaman, 1928	Krbava River, Udbina	Croatia	MT417732	NA
*Paradiplozoon moroccoensis* Koubková, Benovics & Šimková, 2021	*Luciobarbus lepineyi* (Pellegrin, 1939)	Zouala Oasis	Morocco	MT417735	NA
	*L. lepineyi*	Zouala Oasis	Morocco	MT417736	NA
	*L. lepineyi*	Zouala Oasis	Morocco	MT417734	NA
*Paradiplozoon nagibinae* (Gläser, 1965)	*Ballerus ballerus* (Linnaeus, 1758)	Kyjovka River	Czech Republic	AJ563371	NA
*Paradiplozoon opsariichthydis* (Jiang, Wu & Wang, 1989)	*Opsariichthys bidens* Günther, 1873	Zhenjiang, Shaoguan	China	MH794188	NA
*P. opsariichthydis* (Jiang, Wu & Wang, 1989)	*O. bidens*	Zhenjiang, Shaoguan	China	MH794184	NA
*Paradiplozoon pavlovskii* (Bychovsky & Nagibina, 1959)	*Leuciscus aspius* (Linnaeus, 1758)	Morava River	Czech Republic	AJ300714	NA
*Paradiplozoon sapae* (Reichenbach-Klinke, 1961)	*Abramis sapa* (Pallas, 1814)	Morava River	Czech Republic	AJ300713	NA
*Paradiplozoon skrjabini* (Akhmerov, 1974)	*Leuciscus waleckii* (Dybowski, 1869)	Primorsky, Bolshaya Ussurka River	Russia	LC050528	NA
	*Rhynchocypris oxycephalus* (Sauvage & Dabry de Thiersant, 1874)	Takami River	Japan	LC050525	NA
*Paradiplozoon vaalense* Dos Santos, Jansen Van Vuuren & Avenant-Oldewage, 2015	*Labeo umbratus* (Smith, 1841)	Vaal River	South Africa	HG423142	NA
*Paradiplozoon yunnanensis* Fan, Meng, Bai, Xu & Wang, 2018	*Sikukia gudgeri* (Smith, 1934)	NA	China	MW048388	NA
*Sindiplozoon coreius* Cao, Fu, Zou, Li, Wu, Wang, Blazhekovikj-Dimovska & Li, 2022	*Coreius guichenoti* (Sauvage & Dabry de Thiersant, 1874)	NA	China	MW992745	NA
*Sindiplozoon ctenopharyngodoni* (Ling, 1973)	*Ctenopharyngodon idella* (Valenciennes, 1844)	Tangxun Lake	China	DQ098898	NA

Host species, locality of collection and accession number from GenBank for ITS2 sequence of each parasite's species are included. NA, data are not available, newly acquired sequences are marked by asterisks (*).

### Phylogenetic analyses

In addition to newly obtained ITS2 sequences, 49 orthologue sequences of 26 diplozoid species were retrieved from GenBank (Table 2) to infer phylogenetic relationships between diplozoid species from the Middle Eastern region and diplozoid species from other regions. The sequence alignment was built using fast-Fourier transform in MAFFT and applying the G-INS-i iterative refinement algorithm (Katoh *et al*., 2002). All the aligned sequences were trimmed to unify their length. To find the best substitution model, ModelGenerator v. 0.85 (Keane *et al*., 2006) was used, and the best model based on the Bayesian information criterion was selected for further phylogenetic analysis. Intraspecific genetic distances were computed using sequences from the ITS2 region. Uncorrected pairwise distances were calculated in MEGA 11 (Tamura *et al*., 2021). Maximum-likelihood (ML) and Bayesian inference (BI) phylogenetic reconstructions were computed using iQtree v. 2.1 (Nguyen *et al*., 2015) and MrBayes v. 3.2 (Ronquist *et al*., 2012), respectively. The best ML tree was selected from 2000 iterations with support for the branching pattern validating through 2000 bootstrap pseudo-replicates. The BI tree was constructed using the Metropolis-coupled Markov chain Monte Carlo algorithm with two parallel runs comprising four concurrent chains (one cold and three hot) running for 2 000 000 generations, with trees sampled every 100 generations. The initial 30% of trees were discarded as ‘burn-in’, after checking the standard deviation split frequency fell below 0.01. The convergence of evolutionary model parameters was then checked by Tracer v. 1.7.1 (Rambaut *et al*., 2018). The final phylograms were rooted using *Octomacrum europaeum* of Octomacridae, as a representative species of a family phylogenetically proximal to Diplozoidae (Sicard *et al*., 2002).

## Results

### Diversity and infection load of *Paradiplozoon* in the Middle East

A total of four *Paradiplozoon* species were collected from 48 out of 93 investigated host species in three Middle Eastern countries (Table 1). These included *P. bingolensis*, *Paradiplozoon bliccae* and *P. homoion*, as well as *Paradiplozoon koubkovae* n. sp., identified as a new species for science. *Paradiplozoon bingolensis* was collected only from *Cyprinion macrostomum* at two localities in Iraq and *Cyprinion kais* at one locality in Turkey. *Paradiplozoon bliccae* was collected only from cyprinoid species in Turkey and herein, it was recorded on six new host species (Table 1). The 100% prevalence of *P. bliccae* was found on *Luciobarbus kottelati* from the Çine River (Turkey). However, the highest maximum intensity of infection, and mean abundance by *P. bliccae* were recorded on *Petroleuciscus ninae* (i.e. *I* = 1–20, *A* = 2.9) and *Barbus xanthos* (*I* = 1–19, *A* = 5), respectively, at the same locality. *Paradiplozoon homoion* exhibited the widest host range among the collected *Paradiplozoon* species and was found on 36 fish (33 new host records, see Table 1) species belonging to eleven cyprinoid genera. The 100% prevalence of *P. homoion* was recorded on *Alburnoides* aff. *damghani* and *Capoeta buhsei* from Iran; *Alburnus sellal* from Iraq and *Alburnus carianorum*, *Alburnoides kosswigi*, *Barbus tauricus*, *Capoeta damascina* and *Squalius cii* from Turkey. *Paradiplozoon homoion* reached the highest mean abundance on *Alburnus chalcoides* (from Iran) followed by *B. tauricus*, *S. cii*, *A. carianorum* (from Turkey) and *Chondrostoma regium* from Iraq (Table 1). The last species, *P. koubkovae* n. sp., was collected from two localities in Iran and Turkey (Table 1), where only one specimen was found on *Luciobarbus capito* (Iran) and four specimens were collected from *Capoeta capoeta* (Turkey).

### Morphology

#### *Paradiplozoon bliccae* (Reichenbach-Klinke, 1961)

***Description:*** With soft body characters of the species. Attachment apparatus (haptor) consisting of four pairs of clamps and a pair of central hooks lying on ventral side of each haptor. Clamps arranged bilaterally in two parallel longitudinal rows, with their openings directed ventrally. First pair of clamps slightly smaller than other pairs. Each clamp composed of sclerites in configuration typical for species of *Paradiplozoon* (see Fig. 2). Median plate J-shaped, with median groove perforated by lacunae throughout most of its length. Anterior half of median plate distally elongated into rectangular trapeze spur (not trapezoid or fishtail-shaped) and directly connected with anterior jaw. Arches (left, right) of anterior jaw proximally fused together, forming converse T-shaped junction with median plate. Posterior half of median plate with tendon guiding termination comprising a rounded collar-shaped structure distally supported by lightly sclerotized triangular enlargement. Posterior half of median plate connected to arches of posterior jaw by two posterior joining sclerites; proximal posterior joining sclerite twice as long as distal posterior joining sclerite, with median groove. Anterior jaw comprising two (left, right) relatively thin arches; each with median groove perforated by lacunae throughout 0.7 of its length, proximal larger half arched (by its convexity ventrolaterally), distal minor half recurved into wing-shaped part. Wing-shaped part with inwardly directed spur and small joint socket for articulating head of lateral sclerite. Posterior jaw composed of two (left, right) smooth arches; each with thickened convex side and slightly bifurcated end to give bone-like appearance. Two lateral sclerites bilaterally connecting anterior and posterior jaws; each about 0.5–0.75 of length of posterior jaw arch. Central hooks situated in proximity of inner margin of most posterior pair of clamps (i.e. pair of clamp I).

### Remarks

**Figure 3.**
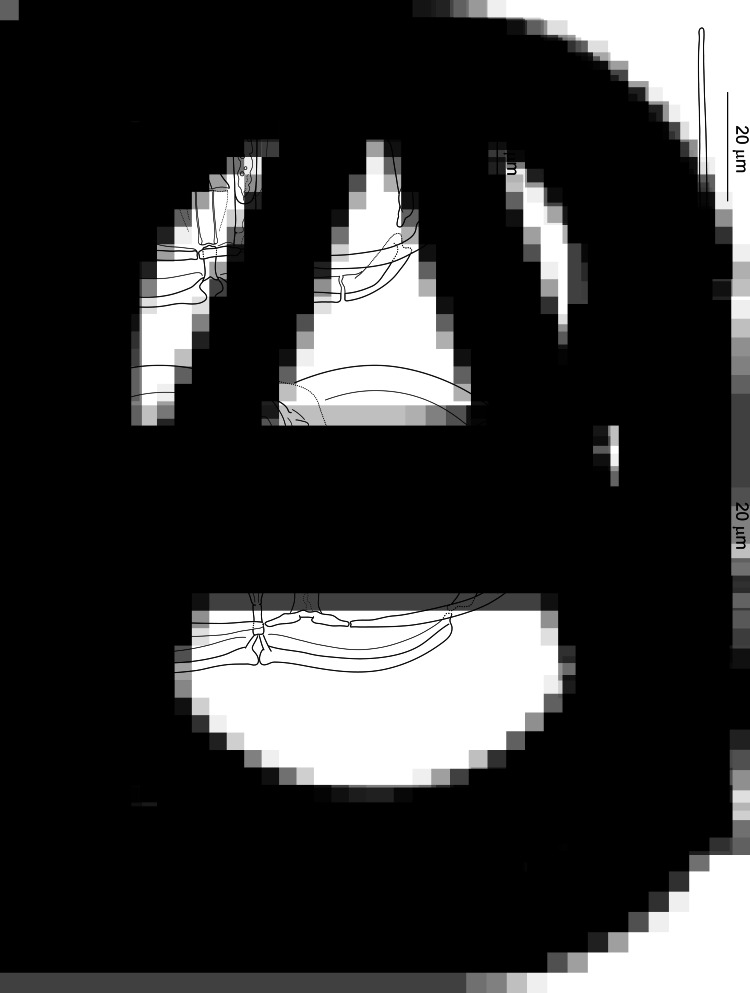
Attachment clamp III and central hook of *Paradiplozoon bliccae* (Reichenbach-Klinke, 1961) from (A, B) *Luciobarbus kottelati* (Turkey) (IPCAS, M-300/4) and (C, D) from *Vimba vimba* (Turkey) (IPCAS, M-300/9).

*Paradiplozoon bliccae* is the only known species of the genus possessing proximally fused arches of anterior clamp jaw, forming converse T-shaped junction with median plate. Two morphological variants of *P. bliccae* were recognized: *P. bliccae* variant A1 (Fig. 3A and B) collected from *B. xanthos*, *Capoeta aydinensis*, *Ladigesocypris ghigii*, *L. kottelati*, *P. ninae*, *Squalius fellowesii* and *Vimba mirabilis*, and *P. bliccae* variant A2 (Fig. 3C and D) collected from *V. vimba*. The uncorrected *P*-distance between *P. bliccae* variants in ITS2 sequences was 0.7%, and the distances within variants A1 and A2 were 2 and 0.5%, respectively (Supplementary Table 2).

Figure 3 provides the morphological differentiation of the two forms: (1) central hooks are generally larger, with significantly longer handle in *P. bliccae* variant A2; (2) T-shaped junction of median plate and anterior jaw (i.e. trapeze spur + proximal ends of arches of anterior jaw) is more massive in *P. bliccae* variant A2 and (3) proximal posterior joining sclerite in *P. bliccae* variant A1 appears to be less sclerotized than that in variant A2. Respective data for variants A1 and A2 (i.e. host and locality records, localities records, measurements and drawings of sclerites) are presented below.

**Figure 4.**
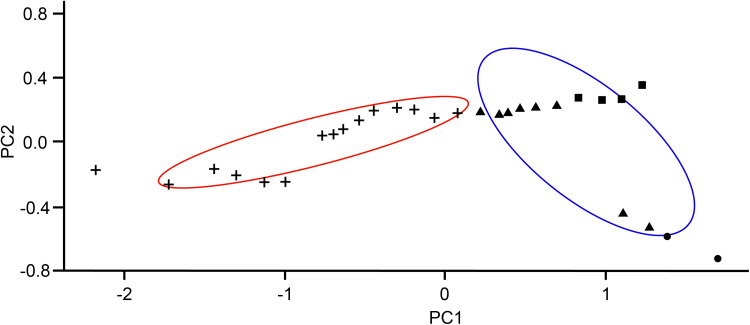
Two variants of *Paradiplozoon bliccae* (A1: blue ellipse and A2: red ellipse) in their morphometrical space based on PCA: (●) *Capoeta aydinensis*, (▴) *Luciobarbus kottelati*, (■) *Squalius fellowesii* and (+) *Vimba vimba*.

The majority of *P. bliccae* specimens collected (i.e. specimens collected from *B. xanthos*, *L. ghigii* and *P. ninae*) were diporpae or were in a recently fused ‘just married’ state without fully developed sclerites in the haptors, and, therefore, were not suitable for investigating intraspecific morphological variability. The remaining specimens were adult specimens of *P. bliccae* of the variant A1. For the study of morphometrical variation among host species, we used the specimens of *P. bliccae* variant A1 parasitizing 3 host species (*C. aydinensis*, *L. kottelati* and *S. fellowesii*) and specimens of *P. bliccae* variant A2 parasitizing *V. vimba*. Using PCA, the first axis (PC1) and the second axis (PC2) contributed 89.50 and 7.52% to explaining the variation in the dataset, respectively. Table 3 shows the correlations between morphometric characteristics and PC1 and PC2. By plotting measurements of the haptoral elements (i.e. clamps and hooks) of *P. bliccae* specimens of variants A1 and A2 in factorial space (PC1 and PC2) (Fig. 4), the two variants were separated according to their geographical distribution (variant A1: south-eastern Aegean drainage area, variant A2: south-western Black Sea).

**Table 3.** First 2 factorial axes produced by PCA were compared to the morphometric parameters using Pearson's correlation coefficients

Parameter	PC1	PC2
Sickle	**0**.**992**	−0.071
Handle	**0**.**983**	0.009
Clamp I width	**0**.**980**	−0.121
Clamp I height	**0**.**987**	−0.093
Clamp II width	**0**.**976**	−0.190
Clamp II height	**0**.**972**	−0.197
Clamp III width	**0**.**992**	−0.021
Clamp III height	**0**.**969**	−0.212
Clamp IV width	**0**.**781**	**0**.**583**
Clamp IV height	**0**.**819**	**0**.**513**

Those parameters for which the correlation coefficients, between original parameters and factorial axes, were statistically significant are given in bold.

***Paradiplozoon bliccae* variant A1** (Fig. 3A and B)

***Hosts, localities and specimens studied (hologenophores):** Barbus xanthos* (locality TRK1) (IPCAS, M-300/2), *C. aydinensis* (locality TRK1) (IPCAS, M-300/3), *L. ghigii* (locality TRK2) (IPCAS, M-300/7), *L. kottelati* (locality TRK1) (IPCAS, M-300/4), *P. ninae* (locality TRK1 and TRK2) (IPCAS, M-300/5), *S. fellowesii* (locality TRK3) (IPCAS, M-300/6) and *V. mirabilis* (locality TRK3) (IPCAS, M-300/8).

***Measurements*** [based on ten adult specimens (20 haptors); in *μ*m]: Size of clamps (width × height): clamp I = 117 (102–128) × 87 (78–93); clamp II = 140 (127–152) × 88 (82–95); clamp III = 139 (106–159) × 84 (71–99) and clamp IV = 128 (100–161) × 80 (67–98). Central hooks: sickle length 22 (21–23); handle length 49 (47–51).

***Paradiplozoon bliccae* variant A2** (Fig. 3C and D)

***Host, locality and specimen studied (hologenophores):** Vimba vimba* (locality TRK5) (IPCAS, M-300/9).

***Description:*** With characters of species [based on measurements of four adult specimens; i.e. eight haptors]: Size of clamps (width × height): clamp I = 117 (109–127) × 81 (70–89), clamp II = 147 (131–164) × 84 (75–101); clamp III = 156 (133–176) × 84 (75–98); clamp IV = 158 (139–170) × 85 (73–111) *μ*m. Central hooks: sickle length 24 (23–26); handle length 58 (54–62).

***Paradiplozoon koubkovae* Řehulková, Nejat and Benovics n. sp.**

***Type host:** Luciobarbus capito* (Güldenstädt, 1773).

***Type locality:*** Ghezel Ozan River, Iran (36°47′16″E, 49°07′14″E).

***Other host and locality:** Capoeta capoeta* Levin, Prokofiev & Roubenyan, 2019, B-20 Canal at Aralık, a drainage of Arax River, Turkey (39°54′26″N, 44°30′28″E).

***Site on host:*** Gill lamellae.

***Specimens studied:*** Holotype, paratype, two hologenophores (IPCAS M-773/1); two vouchers and one hologenophore from *C. capoeta* (IPCAS M-773/2).

***ZooBank registration:*** The Life Science Identifier (LSID) for the new name *Paradiplozoon koubkovae* n. sp. Řehulková, Nejat et Benovics is urn:lsid:zoobank.org:pub:29EED971-8DDB-450E-BF7C-132E534A142F.

***Etymology:*** This species is named after Dr Božena Koubková to honour her contributions to knowledge of diplozoid fauna.

***Description:*** Body X-shaped, comprising two adult individuals fused in permanent copula. Fusion delimiting forebody and hindbody in each specimen; forebody 2750 (*n* = 1) long, 826 (*n* = 1) wide; hindbody 1710 (*n* = 1) long. Tegument with small to inconspicuous annular transverse folds or ridges, less prominent to absent in hindbody.

Forebody pyriform, densely filled with vitelline follicles. Mouth relatively large, crescent-shaped, subterminal on ventral side, opening into buccal cavity. Buccal cavity comprising two muscular buccal suckers located bilaterally close to dorsal surface; each 110 (*n* = 1) in diameter. Pharynx immediately following buccal cavity; 91 (*n* = 1) long and 89 (*n* = 1) wide. Intestinal caecum branched, running posteriorly from pharynx into hindbody, ending blindly close to anterior margin of haptor. Transversal diverticula poorly defined due to vitellarium.

Hindbody without rounded or lobed widening just anterior to the haptor, comprising anteriorly situated reproductive organs lacking sclerotized parts and distally located attachment organ (haptor). Haptor without medial dilatation, composed of four pairs of clamps and one pair of central hooks lying on ventral side of each haptor. Clamps arranged bilaterally in two parallel longitudinal rows. First and fourth pairs of clamps a little smaller than other clamps (Fig. 6A). Size of clamps (based on measurements of six adult specimens/eight haptors; width × length): clamp I = 109 (127–98) × 85 (78–95); clamp II = 114 (138–97) × 82 (98–73); clamp III = 110 (127–97) × 78 (90–70); clamp IV = 96 (116–70) × 71 (85–53). Clamps relatively massive; each with well-developed sclerites in configuration typical for species of *Paradiplozoon* (see Fig. 2). Median plate U-shaped, with median groove on its outer side, perforated by lacunae throughout most of its length. Anterior half of median plate with clearly visible lacunae along the inner edges of the groove, distally enlarged into fishtail-shaped trapeze spur and connected with the anterior clamp jaw by one anterior joining sclerite. Anterior joining sclerite large, cube-shaped (often widened in the proximal part to the shape of an anvil). Posterior half of median plate appearing to be wider than the anterior half, distally slightly narrowing before expanding into tendon guiding termination. Tendon guiding termination comprising collar-shaped part arising subterminally from its outer side and trapezial terminal extension protruding distally from beneath collar (i.e. on inner side of median plate). Posterior half of median plate connected with posterior clamp jaw by two posterior joining sclerites, both tapering distally: one proximal joining sclerite wider than longer, with a knob in middle of its distal side; one distal joining sclerite longer than wider, with conspicuous median groove on its outer surface. Anterior jaw comprising two (left, right) massive arches; each with median groove perforated by lacunae throughout most of its length, proximal larger half arched (by its convexity ventrolaterally), distal minor half recurved into wing-shaped part. Wing-shaped part with well-developed inwardly directed spur and small joint socket for articulating head of the lateral sclerite. Posterior jaw composed of two (left, right) massive smooth arches; each with thickened convex side proximally enlarged into a rounded process. Two lateral sclerites bilaterally connecting anterior and posterior jaw arches; each about 0.5 of length of the posterior jaw arch, with a conspicuous ridge. Central hooks situated in proximity of inner margin of the most posterior pair of clamps (i.e. clamp I); sickle length 27 (29–25; *n* = 8); handle length 57 (50–64; *n* − 8).

### Remarks

**Figure 5.**
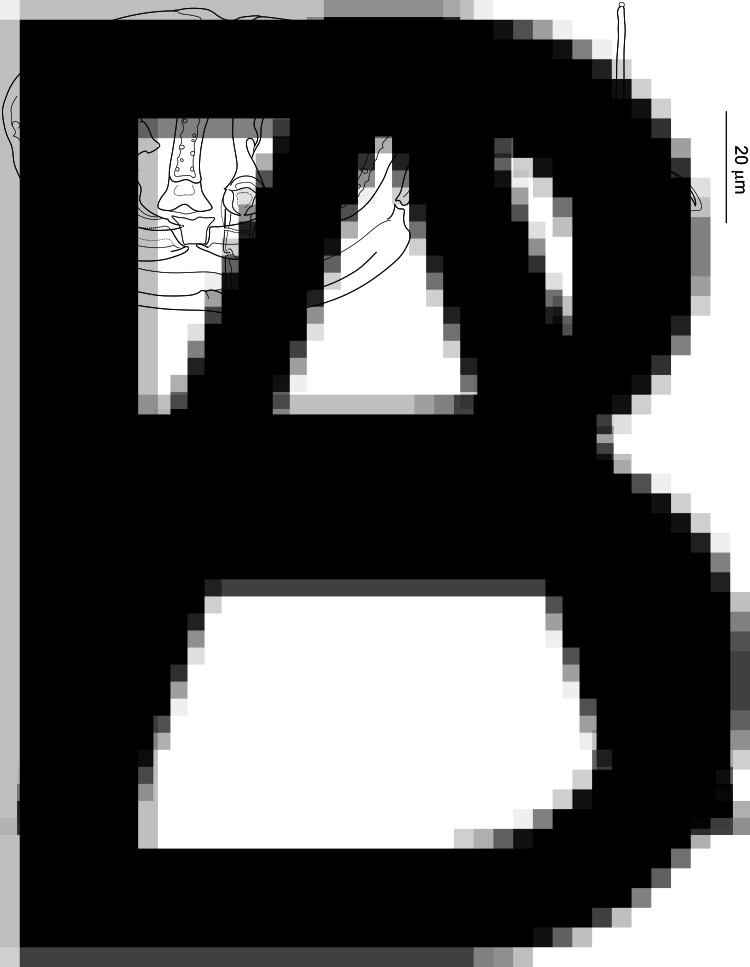
Attachment clamp III (A) and central hook (B) of *Paradiplozoon koubkovae* n. sp. (IPCAS M-773/1) from *Luciobarbus capito* (Iran).

**Figure 6.**
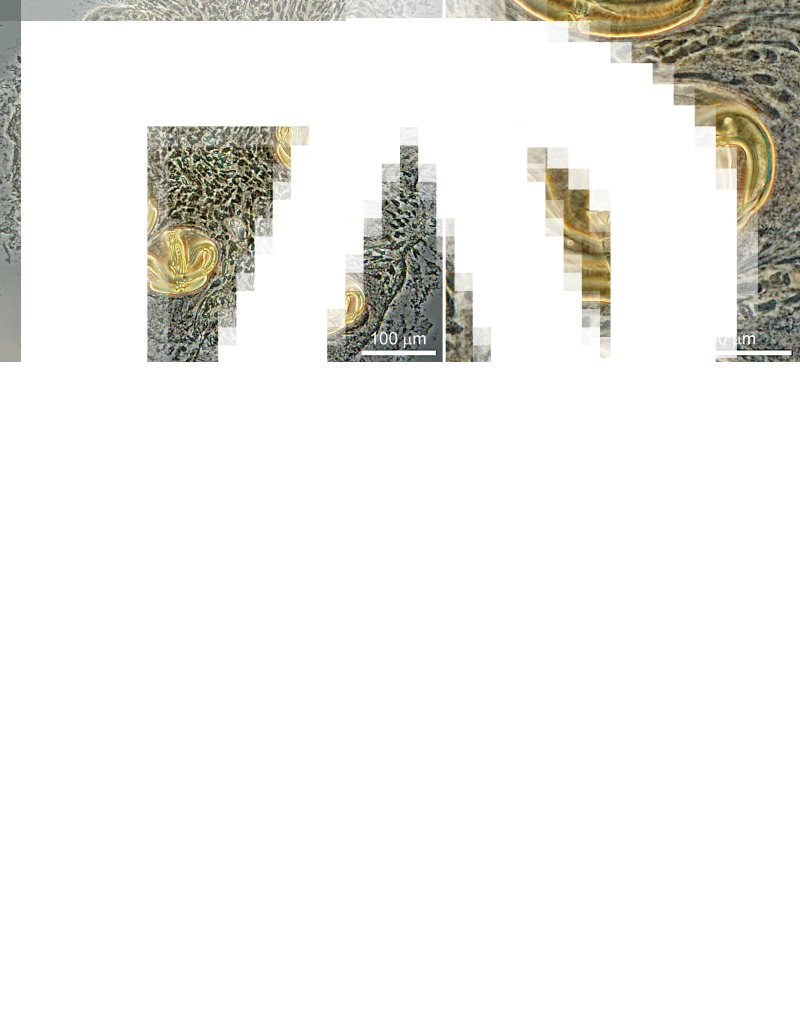
*Paradiplozoon koubkovae* Řehulková, Nejat et Benovics n. sp. from *Luciobarbus capito*. Phase-contrast micrographs of (A) haptor and (B) third clamp.

Although only six haptors were available for morphological study, the diplozoid specimen collected from *L. capito* (Iran) and *C. capoeta* (Turkey) was easily assigned to *Paradiplozoon* on the basis of the absence of prominent dilatation of prehaptoral region in the posterior end of the body. Comparison of the morphological details of clamp sclerites presently known for *P. koubkovae* n. sp. with those of previously described species of *Paradiplozoon* suggests that the specimen reported here represents an undescribed species. The basic configuration of the clamp sclerites indicates that *P. koubkovae* n. sp. (Figs 5 and 6) is morphologically close to *Paradiplozoon vaalense* reported on *Labeo umbratus* in South Africa by Dos Santos *et al*. (2015). On the basis of the morphology of clamp sclerites suggested in the drawings by these authors, the following characters are common to both species: anterior half of the median plate is distally enlarged into a trapeze spur; anterior joining sclerite is relatively massive and cube-shaped; posterior half of median plate is connected with posterior jaw by two posterior joining sclerites; arches of posterior jaw are relatively massive. *Paradiplozoon koubkovae* n. sp. differs from *P. vaalense* by having (1) large, fishtail-shaped trapeze spur (*vs* short, rectangular trapeze spur in *P. vaalense*); (2) proximal posterior joining sclerite longitudinally shorter than distal one (proximal and distal posterior joining sclerites are of similar length in *P. vaalense*); (3) proximal posterior joining sclerite with thickened lateral margins and a medial knob arising from its distal side (compared with pair of match-shaped thickenings passing parallel through middle in *P. vaalense*) and (4) slightly larger central hooks (sickle length 27 and handle length 55 *vs* 19 and 43, respectively, in *P. vaalense*).

Dos Santos *et al*. (2015) stated that in *P. vaalense*, two small additional sclerites are sometimes observed between the anterior joining sclerite and anterior jaw arches. In one attachment clamp of *P. koubkovae* n. sp., the pointed proximal ends of the anterior jaw arches look like two additional sclerites. The two small additional sclerites reported by Dos Santos *et al*. (2015) in *P. vaalense* may actually represent proximal ends of the anterior jaw arches, but further investigation is needed to verify this suggestion.

**Figure 7.**
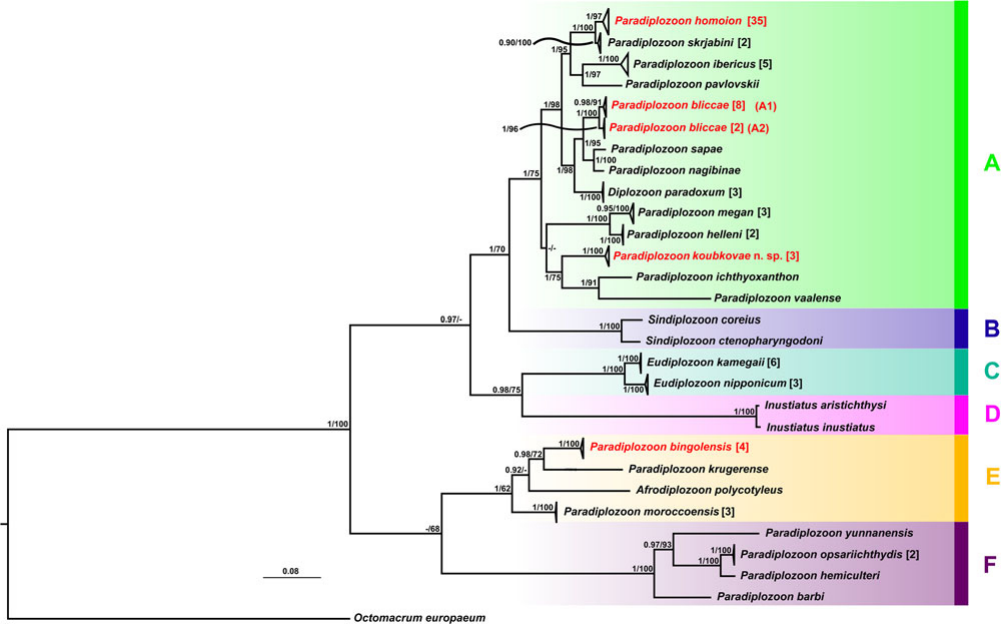
Phylogenetic tree of 95 ITS2 sequences of 27 diplozoid species reconstructed by BI. The numbers at each node represent posterior probabilities and bootstrap support values, resulting from BI and ML analyses, respectively. Dashes indicate posterior probability below 0.70 and bootstrap value below 50. Numbers in the brackets indicate the number of sequences in each collapsed branch. Coloured areas and letters are referred in results.

Comparison of *P. koubkovae* n. sp. with another phylogenetically close species, *Paradiplozoon ichthyoxanthon* in Avenant-Oldewage *et al*. (2014) (see also Fig. 7), was not possible because the original drawing of the clamp sclerites of the latter species lacks the detailed information required for proper species differentiation. However, scanning electron micrographs of the clamp sclerites of *P. ichthyoxanthon* reported by Dos Santos *et al*. (2019) clearly show that the anterior end of the median plate is not extended into the trapeze spur, which is at least one of the features that distinguishes this species from *P. koubkovae* n. sp. However, the scanning electron micrographs of clamp sclerites of *P. ichthyoxanthon* reported by Dos Santos *et al*. (2019) show that the morphological configuration of sclerites connecting the posterior clamp jaws with the median plate is similar to that of *P. koubkovae* n. sp., i.e. comprising proximal posterior joining sclerite with tri-forked distal margin and distal posterior joining sclerite slightly narrowing distally. In addition, unlike *P. vaalense*, both former mentioned species possess relatively large trapeze spur resembling a fish tail. However, *P. koubkovae* n. sp. can be differentiated from *P. ichthyoxanthon* by having the collar-shaped part of the tendon guiding termination with long wings (*vs* short wings in *P. ichthyoxanthon*; cf. Fig. 6I in Dos Santos *et al*., 2019).

### Phylogenetic relationships within the Diplozoidae

The final alignment included 96 ITS2 sequences (including outgroup) and spanned 634 unambiguous nucleotide positions. TVM + G was selected as the best DNA substitution model and used in BI and ML analyses. BI and ML trees showed the same topologies. The resulting BI phylogenetic tree including posterior probabilities and bootstrap support values is presented in Fig. 7. To observe genetic intraspecific variability, the final dataset included all representative sequences for *Paradiplozoon* specimens from the investigated parasite populations in the Middle East (*P. bliccae*, *P. homoion* and *P. bingolensis*). No intraspecific genetic variability was found among *P. bingolensis*. Also, no intraspecific genetic variability was observed among *P. homoion* specimens from various host species, despite this species' remarkable recorded host range. However, substantial intraspecific genetic variability, as well as morphological intraspecific variability, were observed among *P. bliccae* individuals (see remarks on *P. bliccae*, Supplementary Table 2).

Phylogenetic reconstruction revealed six major clades of the Diplozoidae species (Fig. 7). According to the phylogenetic tree, *Paradiplozoon* was polyphyletic, and four *Paradiplozoon* species collected from Middle East cyprinoid species (Fig. 7, species shown in red colour) were placed in two divergent clades. Clade A formed a well-supported monophyletic group and included 13 *Paradiplozoon* species and *Diplozoon paradoxum*. The positions of species in the subclade including *P. megan*, *Paradiplozoon helleni*, *P. koubkovae* n. sp., *P. ichthyoxanthon* and *P. vaalense* were not fully resolved within clade A; however, these five species were divided into two well-supported groups. *Paradiplozoon megan* and *P. helleni* formed a monophyletic group and *P. koubkovae* n. sp. from the Middle East had the sister position to African *P. vaalense* and *P. ichthyoxanthon*. Clades B, C and D each represented different diplozoid genera. *Eudiplozoon* spp. (clade C) and *Inustiatus* spp. (clade D) formed a monophyletic group, which was in sister position to the monophyletic group including *Sindiplozoon* spp. (clade B) and *Paradiplozoon* spp. with *D. paradoxum* (clade A). Clade E included three African species and *P. bingolensis* from the Middle East. Interestingly, *Afrodiplozoon polycotyleus* was in sister position to *P. bingolensis* (the Middle East) and *Paradiplozoon krugerense* (Africa); however, its phylogenetic position was unresolved by bootstrap support values. All the species from clade F were from East Asia. The phylogenetic relationships between clade E, clade F and the big lineage including clades A, B, C and D were unresolved from the current dataset.

## Discussion

A total of 16 diplozoid species were previously reported from 30 Middle Eastern cyprinoid species (Pazooki and Masoumian, 2012; Mhaisen and Abdul-Ameer, 2014; Öktener, 2014; Al-Nasiri and Balbuena, 2016). Herein, we reported four diplozoid species from 48 cyprinoid species and 32 localities in Iran, Iraq and Turkey increasing the total number of diplozoid species in Middle East to 17. Among Middle Eastern diplozoids, *P. bingolensis* is one of the relatively recently described species, originally recorded on *Garra rufa* in Turkey (Civáňová *et al*., 2013). In the present study, this species was also recorded on *C. macrostomum* in Iraq, and *C. kais* in Turkey, expanding its potential distribution range, and adding new host records for this parasite species. Since both *Garra* and *Cyprinion* are phylogenetically related taxa belonging to the highly diversified Cyprinidae (Tan and Armbruster, 2018), we can assume that *P. bingolensis* might represent a generalist parasite species restricted only to phylogenetically related cyprinids, in contrast to the original proposal of its strict host specificity (Civáňová *et al*., 2013). The next diplozoid species reported on Middle Eastern cyprinoids was *P. bliccae*, previously found only in Iraq on *C. macrostomum* and *Cyprinus carpio* (Al-Nasiri, 2009); however, the presence of *P. bliccae* on *C. carpio* is not usual. Surprisingly, we did not find *P. bliccae* on any endemic cyprinoids in Iraq or Iran, although some potential host species (e.g. *C. macrostomum*) were examined in our study. This observation might be influenced by some ecological factors of water environment such are temperature, pH and salinity (i.e. Shah *et al*., 2013; Gilbert and Avenant-Oldewage, 2016*a*, 2016*b*; Mbokane *et al*., 2019; Aydoğdu *et al*., 2020). In Turkey, *P. bliccae* was previously reported on *S. fellowesii* and *Pseudophoxinus burduricus* by İnnal *et al*. (2020), and on *Blicca bjoerkna*, *Scardinius erythrophthalmus*, *Abramis brama* and *V. vimba* from many rivers flowing into the Baltic, Black and Caspian seas (i.e. Moravec, 2001; Pugachev *et al*., 2009).

On the basis of the obtained results, the host range of *P. bliccae* has expanded by six new host species. The third diplozoid species reported from a wide range of Middle East cyprinoids was *P. homoion*. Among Diplozoidae, this species has the widest range of host species concerning European cyprinoids (Benovics *et al*., 2021) and in the Middle East (all main watersheds, i.e. Euphrates and Tigris basins, Namak basin, Aegean Sea basin and Caspian Sea basin), we recorded *P. homoion* on several new hosts of the genera *Capoeta*, *Alburnus*, *Alburnoides* and *Barbus* (Table 1). Other previously reported diplozoid species from the Middle East, i.e. *Paradiplozoon chazarikum* (Mikailov, 1973), and *Paradiplozoon tadzhikistanicum* (Gavrilov & Dzhaliliov, 1965) from Iran (Pazooki and Masoumian, 2012), *E. nipponicum*, *Paradiplozoon amurense* and *Paradiplozoon barbi* from Iraq (Mhaisen and Abdul-Ameer, 2014), and *P. megan*, *P. barbi* and *D. paradoxum* from Turkey (Öktener, 2014) were not found on the cyprinoid species examined in our study, despite our investigation of the same reported catchments or the same host species for the aforementioned diplozoid species. Finally, our study revealed the presence of *P. koubkovae* n. sp. recorded for the first time on *C. capoeta* from Turkey and *L. capito* from Iran (both localities in the Caspian Sea basin).

Morphological intraspecific variability in monogeneans has been highlighted by several studies in relation to water temperature, host body size and/or geographical distribution (e.g. Ergens, 1976; Ergens and Gelnar, 1985; Littlewood *et al*., 1997; Kaci-Chaouch *et al*., 2008; Kmentová *et al*., 2018; Rahmouni *et al*., 2021, 2022). Positive correlations between host body size and parasite body size or clamp size were previously reported also in diplozoids (Matějusová *et al*., 2002). Nevertheless, differences in the shapes of clamps have previously been used to differentiate between diplozoid species (Khotenovsky, 1985*b*; Pugachev *et al*., 2009, Nishihira and Urabe, 2020). For instance, Nishihira and Urabe (2020) utilized the size of the clamps as one of the key features to distinguish between *E. nipponicum* and *Eudiplozoon kamegaii*, although this feature is widely discussed by other authors as taxonomically unimportant (Matějusová *et al*., 2001*b*, 2002, 2004; Přikrylová *et al*., 2018; Benovics *et al*., 2021). In the present study, we used all commonly used measurements of all the haptoral elements, and by removing the effect of host size we evidenced the morphological variability in the clamps of *P. bliccae*, indicating the morphological, and also genetic, differentiation on an interpopulation scale. PCA revealing two variants (A1 and A2) of *P. bliccae* in our study indicates the importance of using the measurements of all haptoral elements in morphological analysis. Considering host phylogeny, the distribution of the two *P. bliccae* variants (A1 and A2) appears to be unrelated to the phylogenetic relationships between the respective host species; however, their diversification seems to be related to the geographical distribution of their host species, i.e. we can assume the vicariant origin of *P. bliccae* variants. While *P. bliccae* specimens from variant A1 parasitized cyprinoids (*S. fellowesii*, *L. kottelati*, *C. aydinensis*, *P. ninae*, *V. mirabilis* and *B. xanthos*) from the Aegean Sea basin, the specimens from variant A2 parasitized cyprinoids (*V. vimba*) from the Black Sea basin.

Previous molecular studies suggested that even geographically distant populations of diplozoid species, more specifically those of *P. homoion* and *E. nipponicum*, exhibit no intraspecific variability in ITS2 (Matějusová *et al*., 2001*b*; Dos Santos and Avenant-Oldewage, 2020). However, Benovics *et al*. (2021) reported genetic intraspecific variability for *Paradiplozoon ibericus* from different cyprinoid species in the Iberian Peninsula (*p-*distance: up to 0.7%) i.e. intraspecific variability was reported in separate parts of this peninsula, and for *P. megan* (*p-*distance: up to 0.2%) from host species in geographically distant regions (i.e. Apennine and Balkan peninsulas). Intraspecific variability in the ITS1 and/or ITS2 regions was also documented for other monogeneans, such as *Gyrodactylus* von Nordmann, 1832 species (Matějusová *et al*., 2001*a*; Nitta and Nagasawa, 2018), or *Dactylogyrus* Diesing, 1850 species (Šimková *et al*., 2004, 2007; Řehulková *et al*., 2021) and thus is also expected in diplozoids. Nishihira and Urabe (2020) used cytochrome *c* oxidase subunit I (COI) and ITS2 to reveal differences between two *Eudiplozoon* species (i.e. *E. nipponicum* and *E. kamegaii*). They suggested that the morphological and molecular divergence between the two species is the result of long-time adaptation to the specific host; thus, *E. nipponicum* on *C. carpio* should be reclassified as *E. kamegaii*, and *E. nipponicum* is strictly host-specific to *Carassius* spp. They documented significant intraspecific variability in COI (i.e. *p*-distance: 2.6–6.1% for *E. kamegaii*) and minor genetic variability in ITS2 (i.e. *p*-distance: 0–0.3% for *E. kamegaii*). The genetic distance found in our study (*P. bliccae*: *p*-distance: 0–0.8%) is greater than that shown for *E. kamegaii* by Nishihira and Urabe (2020). A minor intraspecific genetic variability in ITS2 of *P. bliccae* was reported also by Unal *et al*. (2017); however, the authors did not provide complete sequence data, not *p*-distances, therefore, it is difficult to assess the level of variability. In general, despite the intraspecific genetic variability documented in diplozoids (e.g. Unal *et al*., 2017; Nishihira and Urabe, 2020; Benovics *et al*., 2021), until now, there have been no studies to explore genetic intraspecific variability alongside morphological intraspecific variability.

The idea of the strict host specificity of Diplozoidae species was suggested by Sterba (1957) and further promoted by Bychowsky and Nagibina (1959). However, recent studies have reported several diplozoid species from a wide range of host species in various regions, e.g. *Paradiplozoon hemiculteri* from seven host species in East Asia (Dos Santos and Avenant-Oldewage, 2020); *P. megan* from five host species in Europe; and *P. ibericus* from seven host species in Iberian Peninsula (Benovics *et al*., 2021), which indicates that host specificity in diplozoids is often underexplored and should be interpreted carefully. Additionally, Sicard *et al*. (2001) suggested that host specificity may vary in different diplozoid species, as some species may infect only congeneric host species (i.e. one parasite species infects several host species of the same host genus) or phylogenetically closely related host species at a higher taxonomical level (i.e. host species belonging to the same family), while others infect a wide range of unrelated species. *Paradiplozoon bingolensis* is an example of a diplozoid species infecting the phylogenetically related species of one family i.e. Cyprinidae, and is currently geographically restricted to the Middle East. Benovics *et al*. (2021) even proposed that some diplozoid species exhibit strong geographical specificity, e.g. *P. ibericus* is restricted only to the host species in the Iberian Peninsula and *P. helleni* is limited only to the few endemic species in the Balkans. In the current study, an endemic variant of *P. bliccae* (variant A1, see above) was documented, potentially suggesting an early speciation process in a small geographical region (two separate, yet adjacent, basins), which ultimately could have end in the emergence of new endemic *Paradiplozoon* species in the geographically isolated region.

The host specificity of diplozoids is often considered by some authors as a taxonomically important characteristic (Jiang *et al*., 1985; Al-Nasiri and Balbuena, 2016). As an example, diplozoid specimens recorded in a new region and/or on a new host species are often considered as a new species; e.g. previously described as *Paradiplozoon parabramisi*, *Paradiplozoon diplophyllorchidis* (Jiang *et al*., 1985), *Paradiplozoon parapeleci* (Jiang *et al*., 1985) and *Paradiplozoon jiangxiense* (Jiang *et al*., 1985), which were all revised as synonyms of *P. hemiculteri* (Jirsová *et al*., 2018). All four ‘species’ were collected from the same region (Pearl River basins, China) and each of them parasitized different host species (Dos Santos and Avenant-Oldewage, 2020). In the Middle East in 2016, *P. iraqensis* was described on *C. macrostomum* from Iraq (Al-Nasiri and Balbuena, 2016). This *Paradiplozoon* species shares many similar characteristics (e.g. clamp shapes, body shape, sickle size and handle size) with *P. bingolensis* described earlier on *G. rufa* from Turkey (Civáňová *et al*., 2013). In the present study, *Paradiplozoon* specimens from *C. macrostomum* were found to be morphologically similar to *P. iraqensis* collected in the same area with some minor morphological differences (e.g. clamp size); however, the molecular data revealed that the collected specimens are genetically identical to *P. bingolensis*. Civáňová *et al*. (2013) suggested that the posterior jaw of *P. bingolensis*, which is not divided into medial and lateral sclerites, represents an important taxonomical feature for identification of this species (i.e. distinguishing *P. bingolensis* from the other congeners). The same morphological feature was evidenced also for *P. iraqensis* (Al-Nasiri and Balbuena, 2016) and we can assume that *P. iraqensis* is a morphological variant of *P. bingolensis* (i.e. it should be considered as synonym species). Unfortunately, Al-Nasiri and Balbuena (2016) did not provide any genetic data for *P. iraqensis*, even though the molecular markers for the characterization and identification of diplozoids have been available from the early 2000s (Matějusová *et al*., 2001*b*, 2002, 2004; Přikrylová *et al*., 2018) and various authors have suggested their inclusion in all taxonomical works (e.g. Jirsová *et al*., 2018; Dos Santos and Aventnat-Oldewage, 2020; Benovics *et al*., 2021).

Concerning the molecular phylogenetic reconstruction of diplozoids, previous studies revealed a polyphyly in *Paradiplozoon* (Matějusová *et al*., 2004; Civáňová *et al*., 2013; Jirsová *et al*., 2018; Přikrylová *et al*., 2018; Dos Santos and Avenant-Oldewage, 2020; Benovics *et al*., 2021), which was also revealed in our study. What is noteworthy is the nested position of *A. polycotyleus* and *D. paradoxum* within *Paradiplozoon* species, and the phylogenetic position of *Sindiplozoon* spp., *Eudiplozoon* spp. and *Inustiatus* spp. within Diplozoidae. Our ML and BI phylogenetic reconstructions indicated that diplozoids are divided into two major groups (clades A–D in one group and clades E and F in the other group), in line with previous studies by Dos Santos and Avenant-Oldewage (2020) and Benovics *et al*. (2021). For interpreting the phylogenetic reconstruction, the geographical region of the current distribution of each diplozoid species should be taken into account. African species were included in clades A and E, which reveals the paraphyly of African diplozoid species previously also reported by Dos Santos and Avenant-Oldewage (2016) and Přikrylová *et al*. (2018), indicating that African diplozoid species had most likely two different origins. Benovics *et al*. (2021) hypothesized that the *Paradiplozoon* species of clades E and F most likely dispersed through the Middle East and diversified in Africa or Southeast Asia, respectively, and that species of clade A (in their study, they divided clade A into two clades based on geographical distribution) diversified in Eurasia. Our results showed a larger clade encompassing both Eurasian and African species; however, it is still in line with that of Benovics *et al*. (2021), as the phylogenetic relationships among the subclades are not fully resolved. Moreover, adding *P. koubkovae* n. sp. in the phylogenetic reconstruction revealed the role of the Middle East as a crossroad for cyprinoid hosts as *P. ichthyoxanthon* and *P. vaalense* branched out in Africa and *P. koubkovae* n. sp. has the sister position to them. It should be noted that using additional markers can further contribute to resolving the taxonomy of the Diplozoidae (Dos Santos and Avenant-Oldewage, 2020; Nishihira and Urabe, 2020; Benovics *et al*., 2021). Moreover, addition of missing DNA sequences of other diplozoid species may help to resolve ambiguities in the phylogenetic relationships within the Diplozoidae. As for instance, inclusion of the *P. moroccoensis* sequence into the phylogenetic analysis revealed a topology slightly contradicting that presented by Dos Santos and Avenant-Oldewage (2020).

Finally, Dos Santos and Avenant-Oldewage (2020) suggested the need for major revision of the Diplozoidae. Especially, due to the phylogenetic position of *Afrodiplozoon*, they proposed re-evaluation of the status of two subfamilies of diplozoids. Concurrently with the phylogenetic relationships reported in our study, the previous authors (Dos Santos and Avenant-Oldewage, 2020) also suggested all the species included in clade A represent a monophyletic and divergent taxon to other diplozoid genera, and the species within clade A should be taxonomically reclassified as *Diplozoon* due to consistent grouping of *D. paradoxum* with *Paradiplozoon* spp. This proposal is further supported by the morphology of the clamps in diplozoid species. Species included in clade A have six major parts in the anterior and posterior median sclerite of the clamps, the only exception being *P. ichthyoxanthon* with three parts. *Sindiplozoon* spp. (clade B) and *Eudiplozoon* spp. (clade C) have four parts in the anterior and posterior median sclerite of the clamps, but in different arrangements, while *Inustiatus* (clade D) has five major parts. Species of *Paradiplozoon* in clade F have two parts in the anterior median sclerite of the clamps and different arrangements in the posterior median sclerite to clade E (Pugachev *et al*., 2009; Civáňová *et al*., 2013; Avenant-Oldewage *et al*., 2014; Dos Santos *et al*., 2015; Jirsová *et al*., 2018, 2021; Přikrylová *et al*., 2018; Benovics *et al*., 2021; Cao *et al*., 2022). Considering the morphological similarities, the suggestion of Dos Santos and Avenant-Oldewage (2020) to separate the *Paradiplozoon* and *Afrodiplozoon* species in clade E into two genera needs to be taken with caution, as all the included species of *Paradiplozoon* (clade E) have the same clamp shape.

## Conclusion

Recent knowledge on the diversity, phylogeny and ecology of Diplozoidae is fragmentary, and the resulting knowledge gaps are even more pronounced in the Middle East. This study was designed to fill some of these gaps and, at the same time, examine some issues relating to the current taxonomy of Diplozoidae. Our research suggests that the Middle East may represent the potential region of origin of different lineages of African species of *Paradiplozoon*. The intraspecific variability at the morphological and genetic levels in populations of *P. bliccae* from Middle Eastern cyprinoids suggests that the speciation process might have been promoted even in geographically small regions. In addition, we revealed a lower level of host specificity for currently known *Paradiplozoon* species. Overall, our study highlights the need for taxonomical re-evaluation within the Diplozoidae, based on an integrative morphological, ecological and molecular approach.

## Data Availability

All new DNA sequences of *Paradiplozoon* parasites and host species obtained during this study were deposite ddirectly into NCBI GenBank under accession numbers OP588752–OP588794 and OQ797981-OQ798023 respectively. Type material of new *Paradiplozoon* species was deposited in IPCAS under voucher numbers (IPCAS M-300 and IPCAS M-773).

## Supplementary material

For supplementary material accompanying this paper visit https://doi.org/10.1017/S0031182023000446. click here to view supplementary material
