# Supplementary material for: Diversity, phylogeny and intraspecific variability of Paradiplozoon species (Monogenea: Diplozoidae) parasitizing endemic cyprinoids in the Middle East
Source: Parasitology. 2023 May 9;150(8):705–22. doi: 10.1017/S0031182023000446 (PMC10410381; doi:10.1017/S0031182023000446)
Supplement: Supplementary file 1 [file S0031182023000446sup.zip › S0031182023000446sup002.docx]

Supplementary Table 1: LocID and corresponding localities and year of collection

| LocID | Locality | Country | Coordinates | Year |
| --- | --- | --- | --- | --- |
| IRN1 | Jajrood River, Saeed Abad | Iran | 35°43'40"N 51°41'51"E | 2018 |
| IRN2 | Jajrood River, Lavasan | Iran | 35°48'39"N 51°37'18"E | 2018 |
| IRN3 | Ghare Chay River, Jalayer village, | Iran | 34°53'15"N 50°02'03"E | 2018 |
| IRN4 | Shahrood River, Balastan | Iran | 36°37'02"N 49°32'36"E | 2018 |
| IRN5 | Ghezel Ozan River, Gilvan | Iran | 36°47'16"E 49°07'14"E | 2018 |
| IRN6 | Tajan River, Alavi Kola | Iran | 36°26'15"N 53°09'52"E | 2018 |
| IRN7 | Tajan River, Zarde | Iran | 36°47'55"N 53°08'14"E | 2018 |
| IRQ1 | Suleymania-Dukan, Little Zab | Iraq | 35°52'53"N 45°00'20"E | 2021 |
| IRQ2 | Darbandikhan Lake | Iraq | 35°07'17"N 45°43'50"E | 2019 |
| IRQ3 | Du Choman, Aw-e Shiler River | Iraq | 35°45'49"N 45°27'12"E | 2021 |
| IRQ4 | Dukan Lake | Iraq | 36°10'12"N 44°57'24"E | 2019 |
| IRQ5 | Grdi Go, Zalm Stream | Iraq | 35°18'26"N 45°58'18"E | 2021 |
| IRQ6 | Kani Shok, tributary of Tabin River | Iraq | 35°50'01''N 45°06'16''E | 2021 |
| IRQ7 | Zahrzi, Tabin River | Iraq | 35°48'32''N 45°01'20''E | 2021 |
| TRK1 | Kamışdere stream, near Yatağan | Turkey | 37°20'30"N 28°06'55"E | 2020 |
| TRK2 | Çine River, near Çiftlikköy | Turkey | 37°45'48"N 27°50'03"E | 2020 |
| TRK3 | Kocaalan Deresi | Turkey | 36°57'12"N 28°17'13"E | 2020 |
| TRK4 | Kayırlı Stream, near Yatağan | Turkey | 37°24'36"N 28°06'49"E | 2020 |
| TRK5 | Çifteler | Turkey | 39°20'40"N 31°18'45"E | 2021 |
| TRK6 | East of Barakfakih, Kurutma kanalı, Nilüfer | Turkey | 40°13'27"N 29°18'56"E | 2021 |
| TRK7 | Kütahya | Turkey | 39°22'49"N 30°03'59"E | 2021 |
| TRK8 | Porsuk Tibet | Turkey | 39°46'06"N 30°28'52"E | 2021 |
| TRK9 | Sapanca, inflow to the Sapanca Lake | Turkey | 40°41'54"N 30°14'48"E | 2021 |
| TRK10 | Ardahan, Kura basin | Turkey | 41°06'57"N 42°42'02"E | 2022 |
| TRK11 | Aralik, Aras, Kura basin | Turkey | 39°54'26"N 44°30'28"E | 2022 |
| TRK12 | Yiğitkonağı closest village, Çakır, Kura basin | Turkey | 40°58'01"N 42°35'16"E | 2022 |
| TRK13 | Ölçek, Ölçeksuyu, Kura basin | Turkey | 41°08'01"N 42°51'22"E | 2022 |
| TRK14 | between Otluca and Kasımoğlu, Karasu stream, Van Lake | Turkey | 38°41'25"N 43°23'53"E | 2022 |
| TRK15 | Sinanköy, Akçayır stream (inflow of Batman River), Tigris basin | Turkey | 37°51'56"N 40°59'21"E | 2022 |
| TRK16 | Darköprü, Çelebyian stream, Tigris basin | Turkey | 38°08'02"N 40°49'15"E | 2022 |
| TRK17 | Taşlıburç, Çağ-Çağ stream, Euphrates basin | Turkey | 37°11'56"N 41°18'28"E | 2022 |
| TRK18 | inflow of İyidere, İyidere basin | Turkey | 40°57'39"N 40°22'26"E | 2022 |

s
